# Correspondence

**Published:** 1889-06

**Authors:** George Dock

**Affiliations:** Galveston, Texas


### CORRESPONDENCE.

GALVESTON, TEXAS, June, 1889.

*Editor Daniel's Texas Medical Journal:*

The great success of the last meeting of the State Medical Association, in every respect, must be a source of satisfaction to all who participated in it. At the same time, it should not prevent us from seeking in the future to improve the value of these gatherings in every way. In a spirit purely suggestive, and not at all critical, we submit some ideas formed during and after the meeting.

We take it that these annual meetings have a double aim, medical or scientific, and social, and that each can be cultivated without injury to the other. In order to still further improve the scientific aspect, we believe some such plan as the following could be adopted with advantage: The titles of all papers, or subjects for discussion, to be sent to the chairmen ot sections in whose department they come a month or six weeks before the time appointed for the meeting, together with an estimate of the time necessary for the reading. This time should be limited, subject to extension. These papers should then be arranged in order by the chairman, placing those of similar or allied subjects consecutively. The complete programme of each section should be in the hands of the committee of arrangements at least a month before the meeting, and be published in the JOURNAL, appearing before the same. In this way, members wishing to discuss a paper, and especially those wishing to report cases bearing on any given subject, could have time to arrange their data, and present their views not only more forcibly, but more correctly.

At the meeting, members would know approximately when a given paper would be read, the great advantage of which is obvious.

The absence of any such programme at the San Antonio meeting was keenly felt by many, and was not due entirely to mismanagement on the part of the local committee. It may be supposed we have failed to allow for time for discussion. This would be done by the section chairman in making up each programme, and fiom former experience we can say that by a skillful use of reading by title the time could be very accurately divided. One of the most important factors in the success of a meeting, is the proper selection of section chairmen. In this, not only geographical position and eminence in the profession should be considered, but also enough knowledge of parliamentary tactics to guide the discussion in the right way by bringing out the salient points of papers read, and heading off digressive speakers, and enforcing a time limit. These things are usually followed more or less closely in all scientific assemblies, and we think we are not alone in believing that their adoption would lead to a great increase in the comfort and interest of our annual meeting.

There are other thoughts that arise in this connection, but if those we have presented can serve to excite a healthy discussion we shall feel amply rewarded. I am, sir, with great respect,

Yours truly,

GEORGE DOCK.

